# The Raine study had no evidence of significant perinatal selection bias after two decades of follow up: a longitudinal pregnancy cohort study

**DOI:** 10.1186/s12884-017-1391-8

**Published:** 2017-06-29

**Authors:** Scott W. White, Peter R. Eastwood, Leon M. Straker, Leon A. Adams, John P. Newnham, Stephen J. Lye, Craig E. Pennell

**Affiliations:** 1 School of Women’s and Infants’ Health, The University of Western Australia at King Edward Memorial Hospital, 374 Bagot Road, Subiaco, WA 6008 Australia; 2 Women and Infants Research Foundation, King Edward Memorial Hospital, 374 Bagot Road, Subiaco, WA 6008 Australia; 3 Maternal Fetal Medicine Service, King Edward Memorial Hospital, 374 Bagot Road, Subiaco, WA 6008 Australia; 4 Sir Charles Gairdner Hospital, Hospital Avenue, Nedlands, WA 6009 Australia; 5 School of Medicine and Pharmacology, The University of Western Australia at Sir Charles Gairdner Hospital, Hospital Avenue, Nedlands, WA 6009 Australia; 6 School of Physiotherapy and Exercise Science, Faculty of Health Sciences, Curtin University, Kent Street, Bentley, WA 6102 Australia; 7 The Lunenfeld-Tanenbaum Research Institute Mount Sinai Hospital Joseph and Wolf Lebovic Health Complex 600 University Avenue, Toronto, ON M5G 1X5 Canada

**Keywords:** Raine study, Cohort retention, Bias

## Abstract

**Background:**

Cohort studies may increase or decrease their selection bias as they progress through time. The Western Australian Pregnancy Cohort (Raine) Study has followed 2868 children for over two decades; from fetal into adult life. This paper analyses the cohort over time, assessing potential bias that may come and go with recruitment, retention and loss of participants.

**Methods:**

Linked data from all births in Western Australian over the 3 years the Raine Cohort was recruited were obtained to compare perinatal characteristics and subsequent health outcomes between the Western Australian (WA) contemporaneous birth population and the Raine Cohort at five time points. Perinatal exposure-outcome comparisons were employed to assess bias due to non-participation in Raine Study subsets.

**Results:**

There were demographic differences between the Raine Study cohort and its source population at recruitment with further changes across the period of follow up. Despite these differences, the pregnancy and infant data of those with continuing participation were not significantly different to the WA contemporaneous birth population. None of the exposure-outcome associations were significantly different to those in the WA general population at recruitment or at any cohort reviews suggesting no substantial recruitment or attrition bias.

**Conclusions:**

The Raine Study is valuable for association studies, even after 20 years of cohort reviews with increasing non-participation of cohort members. Non-participation has resulted in greater attrition of socially disadvantaged participants, however, exposure-outcome association analyses suggest that there is no apparent resulting selection bias.

**Electronic supplementary material:**

The online version of this article (doi:10.1186/s12884-017-1391-8) contains supplementary material, which is available to authorized users.

## Background

The Western Australian Pregnancy Cohort (Raine) Study began as a randomised controlled trial evaluating the effects of repeated ultrasound in pregnancy. The Raine Study is now a longitudinal cohort study that has followed the offspring from 18 weeks’ gestation into young adulthood to investigate the early origins of adult disease. Detailed data collection at regular cohort reviews over two decades has seen the Raine Study evolve into a powerful tool for epidemiological and genetic association studies and myriad smaller research projects encompassing a wide range of clinical, para-clinical, and basic science fields.

The Raine Study participants were recruited from the sole tertiary obstetric referral hospital in Western Australia (WA), and surrounding private clinics; WA is the geographically largest and most isolated of the Australian states. Follow up assessments of the cohort over more than two decades has been accompanied by a reduction in participation, as would be expected with any long-term cohort study. It is possible that both the recruitment from a tertiary referral hospital and loss to long-term follow up could influence the extent to which the Raine cohort is representative of the wider Western Australian population; the former potentially increasing the proportion of participants with complicated pregnancies or socioeconomic disadvantage initially recruited and the latter potentially favouring retention of socioeconomically advantaged participants.

Adequate representativeness is clearly important for descriptive studies defining the prevalence of disease within populations, for example, the Global Burden of Disease Initiative [1]. Extrapolating disease prevalence from a non-representative population sample could greatly under- or over-estimate the true population prevalence [2]. Traditional epidemiological practice dictates that cohorts being used for investigation of associations between exposures and outcomes, that is, seeking potential causal relationships, should be reasonably representative of the populations from which they are drawn.

Representative cohorts are desirable as non-representative study groups may introduce bias into observational studies of causal associations, with multiple confounding variables in aetiologically complex conditions being unequally distributed between the population sample and the wider population. For example, previous observational studies demonstrated an apparent association between low antioxidant intake and pre-eclampsia [3–5], the biological plausibility of this link being supported by evidence of oxidative stress in the pathophysiology of established pre-eclampsia [6, 7]. However, the hypothesised reduction in risk with vitamin supplementation, despite early promise [8], was not borne out in subsequent randomised controlled trials among either low-risk [9–11] or high-risk [12–15] women. Meta-analyses of such trials have confirmed similarly poor efficacy [16–19]. It is likely, therefore, that low vitamin status is a surrogate marker for one or more other contributors to pre-eclampsia risk. Statistical adjustment for confounding factors, no matter how apparently thorough, often leaves unidentified residual confounding which, as in this example, is corrected by the randomisation within a controlled trial whereby the study design ensures representativeness between the trial arms [2].

Deliberate non-representative sampling, by contrast, is of scientific value in some situations [2]. For example, twin studies allow the investigation of environmental and epigenetic associations with disease by controlling for genetic variation. “Natural experiments”, such as the Chernobyl reactor disaster, provide insight into rare environmental exposures and subsequent health outcomes. Over-sampling of individuals within minority groups of particular interest allows later subgroup analyses which remain adequately powered without having to recruit a much larger cohort as a whole or to allow for selective attrition of these participants. Achieving representativeness may come at the expense of internal validity, however, a trade-off which has the potential to limit the robustness of inferences of causation, a major objective of observational research [20].

In the context of a longitudinal cohort study, selection bias introduced due to greater loss to follow up among certain subsets of the cohort may influence the assessment of exposure-outcome associations due to evolving differences in the prevalence of exposures, outcomes, or confounding factors between the populations. In particular, if the exposure of interest is associated with the probability of ongoing study participation then the exposure-outcome association may be biased [21] and, therefore, inferences of causation may be flawed. However, previous studies have, by assessing the relative magnitude of known associations in cohorts and their source populations, demonstrated that such bias is likely to be minimal [22, 23].

For large longitudinal studies, in which smaller sub-studies are conducted, the extent of representativeness may be both helpful and a hindrance, depending on the nature of the individual study performed. In such circumstances, therefore, it is particularly important to examine and report the representativeness of the cohort such that external validity may be reliably determined for each study undertaken. Moreover, an assessment of the selection bias resulting from loss to follow up or subgroup analyses should be made.

The aim of this study was to assess perinatal exposure-outcome associations between the Raine Study cohort at multiple time points over the last 20 years and the contemporaneously born Western Australian population.

## Methods

### The Raine study

The recruitment and follow up of the Raine Study has previously been described in detail [24, 25]. In brief, between 1 January 1989 and 31 December 1991, 2900 pregnant women and their fetuses receiving antenatal care at King Edward Memorial Hospital, the sole tertiary referral obstetric hospital in Western Australia, were enrolled prior to 18 weeks’ gestation into a randomised controlled trial investigating the effects of repeated prenatal ultrasound examinations. Liveborn offspring of consenting parents were enrolled into a longitudinal cohort study aimed at assessing the early life origins of adult disease. Follow up assessments at ages 1, 2, 3, 5, 8, 10, 14, 17, 18, and 20 years were undertaken, collecting substantial data concerning, among others, perinatal factors, nutrition, behaviour, neurodevelopment, body composition, cardiovascular and metabolic parameters, musculoskeletal health, mental health, socioeconomic factors, stress responses, genetics and epigenetics, eye health, sleep, and reproductive health.

### Linked data

Linked data were obtained with permission from the Data Linkage Branch of the Department of Health, Western Australia, using the Midwives’ Notification System and Hospital Morbidity database. The Midwives’ Notification System is a compulsory notification of every birth in Western Australia. This system reliably captures all births within hospitals and all births attended by the state-funded home birth programme, together accounting for all Western Australian births apart from a statistically negligible number of unattended births. It records details regarding the mother (age, height, marital status, gravidity, parity, ethnicity, certainty of menstrual dating), the pregnancy (prenatal complications, pre-existing medical comorbidities, onset of labour, labour procedures and complications, and mode of delivery), and the baby (weight, length, head circumference, best estimated gestation, special care nursery admission, and length of hospital stay). This is the only time point at which reliable data are collected regarding the health of every individual in the state. The Hospital Morbidity database captured 303,731 hospital admissions among 107,285 individuals between 1980 and 2010. Of relevance to this study, it contained data regarding neonatal morbidity including jaundice.

Socioeconomic status was described using the publicly available Index of Relative Socioeconomic Disadvantage (IRSD) of the Socioeconomic Indexes for Areas (SEIFA) by the Australian Bureau of Statistics [26]. These data were available either during pregnancy, at birth, or at age 1 year for approximately half of the Raine Study participants who participated in the 14-year follow up assessment. IRSD data were not available for the Western Australian (WA) population subset of mothers giving birth in Perth, so comparisons were made to the entire Western Australian metropolitan population as assessed at the 1991 census (*n* = 1,068,115).

### Cohort subset comparisons

In order to assess how well the Raine Study cohort represents the general Western Australian population on perinatal characteristics, the liveborn offspring of the Raine Study who consented to follow up (the Raine Pregnancy Cohort, *n* = 2863) were compared to the remaining individuals born in Western Australia during the 3 years of recruitment (*n* = 99,141). Comparisons were made between five subsets of the total cohort, including: (i) participants at recruitment (*n* = 2868); (ii) participants of the 5 year follow up assessment (Raine 5-year Cohort, *n* = 2010); (iii) participants of the 20 year follow up assessment (Raine 20-year Cohort, *n* = 1213); (iv) participants with repeated fetal biometry and genome wide single nucleotide polymorphism data (the Raine fetal growth subset, *n* = 1377); and (v) participants who underwent ultrasound screening for non-alcoholic fatty liver disease at age 17 years (NAFLD) (the Raine NAFLD subset, *n* = 879). The latter two subsets are included as examples of deliberately non-representative samples of the entire cohort, having specifically excluded ethnic minorities in order to aid genetic association studies, to allow assessment of any selection bias introduced by deliberate sampling in subgroup analyses.

### Exposure-outcome associations

Comparison methodology was adapted from Nohr et al. [23] who described an approach to assess bias in cohort studies due to non-participation, using known exposure-outcome associations within the study population and the source population. With this method, the strengths of the associations in the two populations are compared by the “relative odds ratio”, whereby a study population with identical strengths of association as its source population will have a relative odds ratio of one. The 95% confidence interval of the relative odds ratio will contain one if there is no statistically significant bias due to non-participation in the study sample for that outcome.

Associations between environmental exposures in pregnancy and adverse perinatal outcomes for various population subsets were assessed using data from the Midwives’ Notification System. In addition, linking records within the Midwives’ Notification System with the Hospital Morbidity dataset allowed associations between epidemiological characteristics of an individual at and after birth.

The perinatal exposures considered in comparisons between the Raine Pregnancy Cohort and the contemporaneously born general WA population were vacuum extraction, low birth weight (less than 2500 g at term), advanced maternal age (greater than 30 years), and preterm birth. The outcome associations examined were spontaneous vaginal delivery and elective caesarean section from the Midwives’ Notification System, and neonatal jaundice from the Hospital Morbidity database as either the primary or additional diagnosis.

More comprehensive data available for the Raine Study subsets allowed the investigation of two further exposure-outcome associations: maternal smoking and low birth weight; and pre-eclampsia and preterm birth.

### Statistics

Statistical analyses were performed with the R statistical software package, version 3.0.1 [27]. Comparisons between groups were made with the Student’s t-test for continuous variables or the Chi-square test for categorical variables. Bonferroni correction for multiple testing suggested a *p*-value of 0.003 as the threshold for significance for exposure-outcome association comparisons between cohorts and a *p*-value of 0.0003 for perinatal characteristics. All assessed variables were effectively normally distributed for the purposes of parametric testing, with the exception of IRSD which was significantly skewed in distribution. Confidence intervals for IRSD were, therefore, calculated using a bootstrapping method of 0.2 trimmed mean and 2000 repetitions for the Raine Study subsets and 100 repetitions for the WA population given the large sample size of the latter group. Comparisons of IRSD between groups were performed by the Wilcoxon rank-sum test.

### Ethics, consent, and permissions

The Health Research Ethics Committee of the Department of Health (Western Australia) granted ethics approval for this study (Project 2010/24, July 2010). The broader Raine Study has ethics approval from The University of Western Australia Human Research Ethics Committee. Informed consent was provided by all participants. Participant assent and parental consent was provided for minors.

## Results

**Fig. 1:**
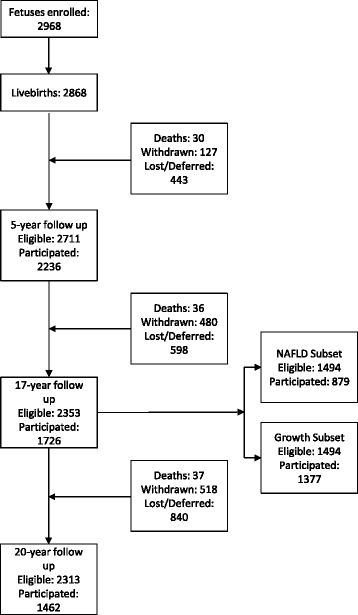
Raine Study participation flow diagram

The Raine Study participant numbers at recruitment and each follow up assessment are described in Fig. 1. The similarities and differences between the Raine Study Pregnancy Cohort at birth, the contemporaneous general WA population, and subsequent the Raine Study follow up and analysis subsets are presented in Table 1 and Additional file 1: Table S1.

**Table 1:** Perinatal demographic comparisons between the general Western Australian population and the evolving Raine Study

	Western Australia		Raine Pregnancy Cohort at birth		Raine 5-year Subset		Raine 20-year Subset		Raine Fetal Growth Subset		Raine NAFLD Subset	
n	99,141		2863		2010		1213		1377		879	
Mothers	Reference											
Age (years)		27.7	=	27.5	+	28.4	+	28.8	+	28.2	+	28.8
Married (%)		89.4%	−	82.1%	−	85.4%	−	86.8%	−	85.7%	−	86.7%
Caucasian (%)		87.4%	+	89.6%	+	90.8%	+	90.8%	+	99.1%^a^	+	99.2%^a^
Nulliparous (%)		39.0%	+	48.1%	+	46.7%	+	47.2%	+	47.6%	+	48.4%
Pregnancies												
Complications (%)		30.0%	+	38.6%	+	37.9%	+	37.4%	+	38.7%	+	46.1%
Caesareans (%)		18.9%	+	21.1%	+	21.7%	+	21.4%	=	20.2%	=	20.4%
Infants												
Birth weight (g)		3344	−	3283	−	3315	−	3316	+	3389	+	3384
Birth length (cm)		49.9	−	48.8	−	49.0	−	49.0	−	49.3	−	49.3
Ponderal index (kg/m^3^)		26.7	+	27.9	+	28.0	+	27.9	+	28.1	+	28.1
Gestation (weeks)		39.1	=	39.0	=	39.1	=	39.2	+	39.3	+	39.3
Nursery admissions (%)		7.6%	+	9.7%	+	9.2%	=	8.5%	=	7.1%	=	7.4%
Socioeconomic status												
IRSD		1022^b^	=	1021	+	1035	+	1046	+	1035	+	1047

^a^The Raine Growth and NAFLD Subsets excluded non-Caucasian participants by design

NAFLD: Non-alcoholic fatty liver disease

IRSD: Index of Relative Socioeconomic Disadvantage. Higher value describes greater socioeconomic advantage

^b^The IRSD for Western Australia includes all Western Australians within the Perth metropolitan area and not just the source population of mainly young metropolitan mothers

Significance defined as *p* < 0.003

+/=/–: Significant increase/no significant difference/significant decrease compared to general Western Australian population

Compared to the WA population of contemporaneous births, the Raine Study mothers were less likely to be married (82% vs 89%, *p* < 0.0001), probably reflecting the relatively lower socioeconomic status of the population referred to a public tertiary antenatal clinic as, at the time of the Raine Study recruitment, ex-nuptial birth was strongly correlated with low socioeconomic status [28] and, indeed, IRSD was lower in unmarried women compared to married women in the Raine Pregnancy Cohort (IRSD 980 vs 1020, *p* < 0.0001). Whilst the Raine Pregnancy Cohort IRSD was not different to the whole WA population IRSD, the whole WA population IRSD included elderly and non-metropolitan groups which suggests IRSD for the source population for the Raine Pregnancy Cohort (mainly young metropolitan mothers) was likely to be higher and the Raine Study average may be elevated by the exclusion of non-participants in the 14-year follow up assessment who were more likely to be socially disadvantaged. Raine Study participants were more likely to be Caucasian (90% vs 87%, *p* < 0.0001), reflecting the English language competency inclusion criterion of the original study. The Raine Study pregnancies were more complicated than the WA population (39% vs 30%, *p* < 0.0001), at least in part due to a higher rate of nulliparous mothers (48% vs 39%, *p* < 0.0001) in whom pre-eclampsia and fetal growth restriction are more common.

Raine Study infants were, on average, 61 g lighter at birth than their WA contemporaries (3283 g vs 3344 g, *p* < 0.0001), again reflecting the higher rate of nulliparity and pregnancy complications. The Raine Study infants were substantially shorter at birth than the WA population (48.8 cm vs 49.9 cm, *p* < 0.0001). This difference in birth length accounts for the observed difference in ponderal index between the Raine Study and WA (27.9 kg/m^3^ vs 26.7 kg/m^3^, *p* < 0.0001). Neonatal nursery admissions were more common in the Raine Study than in WA (9.7% vs 7.6%, *p* < 0.0001), likely due to the higher rate of pregnancy complications and caesarean section in the Raine Study and the immediate availability of nursery facilities in the tertiary hospital setting of the Raine Study.

As the Raine Study has progressed over more than two decades, there have been fewer participants completing all components of cohort reviews: 1213 participants completed the 20-year assessment representing 42% of the original cohort. In general, those who continue to engage with cohort assessments are similar to the original Raine Pregnancy Cohort on perinatal characteristics. Participants who remained engaged were more likely to have older mothers who were married at recruitment than those not participating in childhood and adult reviews. Socioeconomic indices showed greater advantage among those retained to follow up. This reflects selective attrition of those from socioeconomically disadvantaged backgrounds. The pregnancy and infant data of those with continuing participation was not significantly different to the WA contemporaneous birth population (detailed data presented in Additional file 1: Table S1.

The Raine Growth Subset and Raine NAFLD Subset were compared to the Raine Pregnancy Cohort by assessing their perinatal characteristics. Similar to those completing the five and 20-year cohort reviews, these two subsets share the same loss of socioeconomically disadvantaged participants; both had a greater proportion of older mothers (28.2 years and 28.8 years at the Growth and NAFLD subsets, respectively, vs 27.5 years at birth) who were married at recruitment (86% and 87% at the five and 20-year follow up, respectively, vs 82% at birth) than the original cohort. Again, the pregnancy and infant data of those in the subsets were not significantly different to the cohort at recruitment.

### Exposure-outcome associations

The associations between epidemiological factors or environmental exposures and health outcomes are presented in Tables 2, 3, and 4. Among the general contemporaneous births WA population, birth by vacuum extraction was significantly associated with an increased risk of neonatal jaundice (odds ratio 1.42, 95% CI 1.25 to 1.62, *p* < 0.001), a recognised complication of this obstetric intervention. A similar increase was observed in the Raine Pregnancy Cohort (odds ratio 2.11, 95% CI 1.38 to 3.23, *p* < 0.001), as well as in the Raine Study subsets, although this trend did not maintain statistical significance in the smaller subsets (Table 2). The relative odds ratio confidence intervals for this association all included one, not suggestive of significant selection bias upon these particular outcomes (Table 3).

**Table 2:** Exposure-disease associations within the general Western Australian population and the Raine Study subsets. Data presented as odds ratio, 95% confidence interval, and *p*-value

	Western Australia	Raine Pregnancy Cohort at birth	Raine 5-year Subset	Raine 20-year Subset	Raine Growth Subset	Raine NAFLD Subset
Vacuum extraction vs. neonatal jaundice	1.42	2.11	1.94	1.87	1.22	1.21
	(1.25, 1.62)	(1.38, 3.23)	(1.15, 3.28)	(0.93, 3.77)	(0.54, 2.71)	(0.56, 2.58)
	<0.001	<0.001	0.013	0.079	0.634	0.629
Low birth weight vs. spontaneous vaginal delivery	0.73	0.77	0.73	0.72	0.85	0.71
	(0.71, 0.75)	(0.65, 0.91)	(0.59, 0.90)	(0.55, 0.93)	(0.65, 1.11)	(0.54, 0.93)
	<0.001	0.002	0.003	0.014	0.234	0.014
Maternal age > 30y vs. elective caesarean section	1.87	1.89	2.07	2.89	1.92	2.51
	(1.79, 1.95)	(1.48, 2.41)	(1.54, 2.77)	(1.91, 4.37)	(1.33, 2.78)	(1.66, 3.79)
	<0.001	<0.001	<0.001	<0.001	<0.001	<0.001
Preterm birth vs. neonatal jaundice	4.30	4.58	7.48	8.98	9.84	7.88
	(3.90, 4.73)	(3.59, 5.84)	(5.33, 10.51)	(5.55, 14.53)	(5.99, 16.16)	(4.90, 12.70)
	<0.001	<0.001	<0.001	<0.001	<0.001	<0.001
Maternal smoking vs. low birth weight	NA	1.87	1.86	1.97	1.87	2.12
		(1.51, 2.31)	(1.42, 2.44)	(1.39, 2.79)	(1.34, 2.62)	(1.48, 3.05)
		<0.001	<0.001	<0.001	<0.001	<0.001
Pre-eclampsia vs. preterm birth	NA	1.89	1.64	2.04	1.76	1.81
		(1.30, 2.73)	(1.20, 2.24)	(1.35, 3.09)	(1.68, 2.66)	(1.18, 2.76)
		<0.001	0.002	<0.001	0.007	0.006

**Table 3:** Comparison of exposure-disease associations between the general Western Australian population and the evolving Raine Study. Data presented as relative odds ratios and 95% confidence intervals

	Western Australia	Raine Pregnancy Cohort at birth	Raine 5-year Subset	Raine 20-year Subset	Raine Growth Subset	Raine NAFLD Subset
Vacuum extraction vs. neonatal jaundice	Reference	1.48	1.37	1.32	0.86	1.24
		(0.78, 2.20)	(0.59, 2.14)	(0.39, 2.25)	(0.13, 1.59)	(0.40, 2.09)
Low birth weight vs. spontaneous vaginal delivery		1.05	1.00	0.99	1.17	0.98
		(0.94, 1.17)	(0.87, 1.14)	(0.82, 1.15)	(0.97, 1.37)	(0.81, 1.14)
Maternal age > 30y vs. elective caesarean section		1.01	1.11	1.54	1.03	1.34
		(0.61, 1.41)	(0.59, 1.62)	(0.57, 2.52)	(0.44, 1.62)	(0.50, 2.19)
Preterm birth vs. neonatal jaundice		1.07	1.74	2.09	2.29	1.83
		(0.16, 1.97)	(0.00, 3.86)	(0.00, 5.50)	(0.00, 6.12)	(0.00, 4.80)

**Table 4:** Comparison of exposure-disease associations within Raine Study subsets and the Raine Pregnancy Cohort at birth. Data presented as relative odds ratios and 95% confidence intervals

	Raine Pregnancy Cohort at birth	Raine 5-year Subset	Raine 20-year Subset	Raine Growth Subset	Raine NAFLD Subset
Vacuum extraction vs. neonatal jaundice	Reference	0.92	0.89	0.58	0.57
		(0.61, 1.22)	(0.29, 1.48)	(0.28, 0.87)	(0.22, 0.92)
Low birth weight vs. spontaneous vaginal delivery		0.95	0.84	1.11	0.93
		(0.89, 1.02)	(0.82, 1.05)	(0.95, 1.27)	(0.80, 1.05)
Maternal age > 30y vs. elective caesarean section		1.09	1.53	1.02	1.33
		(0.77, 1.42)	(0.64, 2.42)	(0.59, 1.48)	(0.59, 2.07)
Preterm birth vs. neonatal jaundice		1.63	1.96	2.15	1.72
		(0.00, 3.55)	(0.00, 5.24)	(0.00, 5.87)	(0.00, 4.54)
Maternal smoking vs. low birth weight		1.00	1.06	1.00	1.14
		(0.74, 1.25)	(0.60, 1.51)	(0.61, 1.40)	(0.60, 1.67)
Pre-eclampsia vs. preterm birth		0.87	1.08	0.93	0.96
		(0.48, 1.25)	(0.71, 1.46)	(0.82, 1.05)	(0.73, 1.18)

Similarly, low birth weight was associated with a significantly decreased likelihood of birth by spontaneous vaginal delivery than babies of normal birth weight, with small fetuses 25% less likely to be born by spontaneous vaginal delivery (Table 2). This observation was consistent between the general contemporaneous births WA population (odds ratio 0.73, 95% CI 0.71 to 0.75, *p* < 0.001) and the Raine Pregnancy Cohort (odds ratio 0.77, 95% CI 0.65 to 0.91, *p* = 0.002) as well as in the Raine Study subsets, although again this trend did not maintain statistical significance in the smallest subsets. The relative odds ratio confidence intervals for this association all included one, again suggesting that the Raine Study is representative of its source population for this outcome (Table 3).

Maternal age greater than 30 years was associated with a significant increase in elective caesarean delivery: approximately double the rate in younger mothers across the general contemporaneous births WA population and all Raine Study subsets. A similar pattern was demonstrated for the association between preterm birth and neonatal jaundice. This consistency of associations across the various cohorts leads to relative odds ratios close to one, and confidence intervals including one, failing to demonstrate evidence of significant selection bias for these associations (Table 3).

The relative odds ratios for associations between preterm birth and neonatal jaundice in the five- and 20-year subsets deviated substantially from one (Table 2), and it may be that this association is particularly sensitive to the effects of cohort attrition. The reasons for this sensitivity are not entirely clear, however a contribution may relate to loss of estimate precision with the reduction in sample size due to cohort attrition and subgroup analysis. However, the confidence intervals still included one suggesting a lack of significant selection bias for this association.

These four exposure-outcome associations, with the two additional comparisons of maternal smoking during pregnancy and low birth weight and of pre-eclampsia and preterm birth, were compared between the Raine Pregnancy Cohort and the subsequent subsets of the Raine Study (Table 4). The majority of analyses suggested that the Raine Study subsets were representative of the original cohort at birth for these outcomes, despite the apparent demographic differences which occurred among the cohort during the course of follow up in association with some attrition of socioeconomically disadvantaged participants during the course of follow up. Only the associations between vacuum extraction and neonatal jaundice suggested that bias may have been introduced by the subgroup analysis of the Raine Growth and NAFLD Subsets, with relative odds ratio confidence intervals excluding one for these comparisons.

## Discussion

Assessing the perinatal characteristics of the Raine Pregnancy Cohort demonstrates some statistically and potentially clinically significant demographic differences between the cohort and its source population of contemporaneously born Western Australians. Moreover, there have been some further demographic changes in the cohort as it has evolved over the more than two decades of the study through non-participation in follow up and subgroup analyses. These demographic differences have the potential to influence the capacity of the Raine Study to demonstrate exposure-outcome associations and to diminish the external validity of the findings of the Raine Study. It is important, however, to recognise that statistically significant demographic non-representativeness does not necessarily result in significant selection bias, and this should be assessed independently of demographic characteristics.

One method of assessing bias in cohort studies due to non-participation is to compare exposure-outcome associations within the study population and the source population. When this method was applied to the Raine pregnancy cohort, and the five- and 20-year follow up cohorts, the exposure-outcome association comparisons were all not significantly different to the exposure-outcome odds ratios in the WA general population of contemporaneous births. These data suggest that any demographic differences that exist between the WA population and the Raine study cohorts have not introduced significant bias into the Raine study, suggesting the Raine study findings can be generalised to the WA population.

In general, any selection bias due to non-participation in follow up assessments appears not to have any substantial effect on the representativeness of the evolving Raine Study for the outcomes assessed. It must be acknowledged that this study is unable to exclude an effect of selection bias for all possible outcomes, however, the consistency of null findings suggests that cohort non-participation does not introduce systematic bias into the findings of the Raine Study and the generalizability of findings to the source population.

Of the six exposure-outcome associations tested, only one suggested that significant bias may be present when comparing the original Raine Pregnancy Cohort to the genetic analysis subsets of Growth and NAFLD. This is not entirely unexpected, as these subsets were specifically designed to be ethnically homogeneous and this, by design, has introduced non-representativeness to these subsets. This deliberate homogeneity is required for genetic analyses, as ethnic genetic heterogeneity has the capacity to cloud the often subtle associations with single genetic variants: this is an example of a study design where population representativeness is neither necessary nor helpful. This potential source of bias must, however, be taken into account when extrapolating the findings of these particular subgroup analyses to the general population, and similar caution should be applied to other analyses from deliberately non-representative subgroups.

One of the strengths of the Raine Study, when compared to contemporaneous cohort studies, is its high cohort retention over the last 20 years despite the frequency of cohort reviews and the significant time commitment required of participants at each of these reviews. This is largely due to the substantial investment in cohort management, including participant involvement and the use of emerging social media technologies in cohort engagement. There has, however, been some selective attrition of participants from socioeconomically disadvantaged perinatal backgrounds who may face greater challenges to ongoing participation and are more difficult to engage [29]. How these perinatal socioeconomic changes reflect changes in subsequent assessments of socioeconomic status should be evaluated in further research.

Cohort studies are expensive to recruit and even more expensive to regularly review for decades; the Raine pregnancy cohort cost more than AUD$1 M to recruit and more than AUD$25 M has been spent on cohort reviews. The data in this study demonstrate that this money has been well spent because even though non-participation in cohort reviews has increased over time, those who continue to participate are a non-biased group representative of the WA population. Providing that attrition rates are limited, the value of this cohort will increase as they age due to the unique nature of the Raine pregnancy cohort with its dense data collected during pregnancy and the hundreds of thousands of phenotype measures available for analyses.

This work is potentially limited to its confinement to ssessment of selection bias within zexposures and outcomes within the perinatal period. Selection bias may exist in later outcomes which have not been assessed with this approach. Available data did not permit accurate assessment of health outcomes at later life stages due to the relative infrequency of morbidity severe enough to be captured by hospital admissions. Alternative approaches to assessment of selection bias, such as multiple imputation, are likely to be of benefit to the Raine Study and this will be a target of future research.

## Conclusions

The Raine Study cohort had, at recruitment, slightly different perinatal demographic characteristics to the general contemporaneous births WA population. As the Raine Study cohort has evolved over time there has been a shift in demographic characteristics back towards the source population which may or may not have made the cohort more representative depending on the unknown influences of unmeasured potential factors. Neither the limited perinatal differences at recruitment nor at later follow up assessments appear to have resulted in significant selection bias. Subgroup analyses, however, may be somewhat less representative, limiting the external validity of the findings of the Raine Study to broader populations, especially to non-Caucasian populations.

## Additional file

Additional file 1: Table S1. Perinatal characteristics of the Raine Study Birth Cohort, the contemporaneous Western Australian population, and the evolving Raine Study. (PDF 194 kb)

## Abbreviations

AUD: Australian dollars
IRSD: Index of Relative Socioeconomic Disadvantage
NAFLD: Non-alcoholic fatty liver disease
SEIFA: Socioeconomic Indexes for Areas
WA: Western Australia
