# Supplementary material for: The Raine study had no evidence of significant perinatal selection bias after two decades of follow up: a longitudinal pregnancy cohort study
Source: BMC Pregnancy Childbirth. 2017 Jun 29;17:207. doi: 10.1186/s12884-017-1391-8 (PMC5492127; doi:10.1186/s12884-017-1391-8)
Supplement: Additional file 1: Table S1. — Perinatal characteristics of the Raine Study Birth Cohort, the contemporaneous Western Australian population, and the evolving Raine Study. (PDF 194 kb) [file 12884_2017_1391_MOESM1_ESM.pdf]

Supplemental Table 1. Perinatal characteristics of the Raine Study Birth Cohort, the contemporaneous Western Australian population, and the evolving Raine Study.

	Cohort	*Western Australia*[a]	*Raine Pregnancy Cohort* [b]		*Raine Study 5 Year* [c]			*Raine Study 20-year* [d]			*Raine Study Growth* [e]			*Raine Study NAFLD* [f]		
	Total n	*99 141*	*2863*		*2010*			*1213*			*1377*			*879*		
		Mean (95% CI) or n (%)	*Mean (95% CI) or n (%)*	*p (WA)*	*Mean (95% CI) or n (%)*	*p (WA)*	*p (Raine)*	*Mean (95% CI) or n (%)*	*p (WA)*	*p (Raine)*	*Mean (95% CI) or n (%)*	*p (WA)*	*p (Raine)*	*Mean (95% CI) or n (%)*	*p (WA)*	*p (Raine)*
Mother	**Age** (y)	27.7 (27.65, 27.71)	27.5 (27.33, 27.76)	0.229	28.4 (28.10, 28.61)	<0.0001	<0.0001	28.8 (28.46, 29.10)	<0.0001	<0.0001	28.2 (27.93, 28.54)	<0.0001	<0.0001	28.8 (28.41, 29.19)	<0.0001	<0.0001
	**Marital status:**															
	Married	88 597 (89.4%)	2 351 (82.1%)	<0.0001	1 716 (85.4%)	<0.0001	<0.0001	1 053 (86.8%)	<0.0001	<0.0001	1 177 (85.7%)	<0.0001	<0.0001	762 (86.7%)	0.012	0.002
	Not married	10 544 (10.6%)	512 (17.9%)		294 (14.6%)			160 (13.2%)			196 (14.3%)			117 (13.3%)		
	**Ethnicity:**															
	Caucasian	86 607 (87.4%)	2 566 (89.6%)	<0.0001	1 826 (90.8%)	<0.0001	0.129	1 102 (90.8%)	<0.0001	0.204	1 360 (99.1%)	<0.0001	<0.0001	872 (99.2%)	<0.0001	<0.0001
	Non-Caucasian	12 534 (12.6%)	297 (10.4%)		184 (9.2%)			111 (9.2%)			13 (0.9%)			7 (0.8%)		
	**Parity:**															
	0	38 579 (39.0%)	1 377 (48.1%)	<0.0001	939 (46.7%)	<0.0001	0.738	572 (47.2%)	<0.0001	0.719	653 (47.6%)	<0.0001	0.991	425 (48.4%)	<0.0001	0.972
	1	32 393 (32.7%)	821 (28.7%)		607 (30.2%)			368 (30.4%)			395 (28.8%)			248 (28.2%)		
	2	17 652 (17.8%)	425 (14.8%)		297 (14.8%)			174 (14.4%)			208 (15.2%)			136 (15.5%)		
	3	6 660 (6.7%)	167 (5.8%)		119 (5.9%)			70 (5.8%)			82 (6.0%)			52 (5.9%)		
	4	2 365 (2.4%)	50 (1.7%)		35 (1.7%)			21 (1.7%)			22 (1.6%)			13 (1.5%)		
	5	788 (0.8%)	14 (0.5%)		7 (0.3%)			6 (0.5%)			6 (0.4%)			3 (0.3%)		
	≥ 6	584 (0.6%)	9 (0.3%)		5 (0.2%)			1 (0.1%)			3 (0.2%)			2 (0.2%)		
	**Socioeconomic status:** [g]															
	IRSD	1022 (1022, 1022) [h]	1021 (1014, 1025)	0.766	1035 (1025, 1037)	<0.001	0.003	1046 (1033, 1047)	<0.001	<0.001	1035 (1027, 1042)	<0.001	0.001	1047 (1034, 1049)	<0.001	<0.001
Pregnancy	**Pregnancy complications**	29 771 (30.0%)	1 106 (38.6%)	<0.0001	762 (37.9%)	<0.0001	0.310	454 (37.4%)	<0.0001	0.150	531 (38.7%)	<0.0001	0.989	405 (46.1%)	<0.0001	0.378
	**Mode of delivery:**															
	Spontaneous vertex	63 077 (63.6%)	1 750 (61.1%)	<0.0001	1 218 (60.6%)	<0.0001	0.477	723 (59.6%)	<0.0001	0.062	857 (62.4%)	0.073	0.090	465 (60.1%)	0.242	0.806
	Breech	1083 (1.1%)	33 (1.2%)	0.828	17 (0.8%)	0.338	0.361	11 (0.9%)	0.627	0.592	10 (0.7%)	0.241	0.255	7 (0.8%)	0.498	0.477
	Instrumental	17 320 (17.5%)	508 (17.7%)	0.655	355 (17.7%)	0.804	0.958	230 (19.0%)	0.097	0.266	239 (17.4%)	0.969	0.772	158 (18.0%)	0.728	0.915
	Caesarean section	18 744 (18.9%)	605 (21.1%)	<0.0001	437 (21.7%)	<0.0001	0.523	260 (21.4%)	0.005	0.809	277 (20.2%)	0.137	0.370	248 (20.4%)	0.291	0.659
Child	**Weight** (g)	3 344 (3340, 3348)	3 283 (3260, 3306)	<0.0001	3 315 (3289, 3341)	0.034	0.077	3 316 (3284, 3348)	0.088	0.109	3 389 (3359, 3418)	0.003	<0.0001	3 384 (3348, 3420)	0.031	<0.0001
	**Length** (cm)	49.9 (49.9, 50.0)	48.8 (48.7, 49.0)	<0.0001	49.0 (48.9, 49.1)	<0.0001	0.122	49.0 (48.9, 49.2)	<0.0001	0.046	49.3 (49.2, 49.4)	<0.0001	<0.0001	49.3 (49.1, 49.5)	<0.0001	<0.0001
	**Ponderal index** (kg/m^3)	26.7 (26.7, 26.7)	27.9 (27.7. 28.0)	<0.0001	28.0 (27.8, 28.1)	<0.0001	0.224	27.9 (27.8, 28.1)	<0.0001	0.507	28.1 (28.0, 28.3)	<0.0001	0.015	28.1 (27.9, 28.3)	<0.0001	0.025
	**Head circumference** (cm)	34.4 (34.4, 34.4)	34.5 (34.4, 34.5)	0.120	34.6 (34.5, 34.7)	<0.0001	0.060	34.6 (34.5, 34.7)	<0.0001	0.024	34.7 (34.7, 34.8)	<0.0001	<0.0001	34.8 (34.7, 34.9)	<0.0001	<0.0001
	**Gestation** (weeks)	39.1 (39.1, 39.1)	39.0 (38.9, 39.1)	0.045	39.1 (39.0, 39.2)	0.857	0.129	39.2 (39.1, 39.3)	0.098	0.010	39.3 (39.2, 39.4)	<0.0001	<0.0001	39.3 (39.2, 39.4)	0.0004	<0.0001
	**Nursery admission**	7 545 (7.6%)	277 (9.7%)	<0.0001	184 (9.2%)	0.005	0.529	103 (8.5%)	0.230	0.209	97 (7.1%)	0.442	0.003	65 (7.4%)	0.860	0.047

[a] *Western Australia*: all individuals born in Western Australia contemporaneously to the Raine Study (1 January 1989 to 31 December 1992), excluding the Raine Study participants

[b] *Raine Pregnancy Cohort*: all liveborn offspring participants of the Western Australian Pregnancy Cohort (Raine) Study

[c] *Raine 5-year Subset*: Raine Study participants who took part in the 5 year follow up assessment

[d] *Raine 20-year Subset*: Raine Study participants who took part in the 20-year follow up assessment

[e] *Raine Growth Subset*: Raine Study participants with at least one Caucasian parent, who were liveborn, singletons, unrelated, without significant congenital anomalies, and who consented to genetic association studies

[f] *Raine Non-alcoholic Fatty Liver Disease Subset:* Those members of the Raine Study Growth Subset who underwent abdominal ultrasound assessment of non-alcoholic fatty liver disease features at age 17 years

[g] Socioeconomic status described by Socioeconomic Indexes For Areas (SEIFA, Australian Bureau of Statistics): IRSD = Index of Relative Socioeconomic Disadvantage. Greater values represent greater socioeconomic advantage. Confidence intervals derived by bootstrapping method with mean trimmed by 0.2 and 2000 repetitions.

[h] IRSD data were not available for the Western Australian subset of mainly young metropolitan mothers, instead data for the entire Western Australian metropolitan area at the 1991 census is provided.

p (Raine) and p (WA) represent p-values for comparisons against the Raine Study Birth Cohort and the Western Australian population.

na = not available/applicable
